# Pro-sociality and strategic reasoning in economic decisions

**DOI:** 10.3389/fnbeh.2015.00140

**Published:** 2015-05-28

**Authors:** Benito Arruñada, Marco Casari, Francesca Pancotto

**Affiliations:** ^1^Pompeu Fabra University and Barcelona Graduate School of EconomicsBarcelona, Spain; ^2^Department of Economics, Università di BolognaBologna, Italy; ^3^Department of Comunication and Economics, Università degli Studi di Modena e Reggio EmiliaReggio Emilia, Italy

**Keywords:** experiments, homo oeconomicus, social orientation, bounded rationality JEL codes: C91 C92 D63

## Abstract

We study the relationship between pro-social preferences and strategic reasoning. These aspects are typically studied separately but little is known about their joint distribution. In an experiment, for each participant we elicit individual concerns toward pro-sociality—inequality aversion and efficiency—as well as the number of steps of reasoning through a guessing game. We report that self-regarding and pro-social participants exhibit similar levels of strategic reasoning, which supports the view that pro-sociality and strategic reasoning can be studied independently.

## 1. Introduction

Modeling motivations in economic choices and bounded rationality have both been at the center of a long lasting debate. The canonical model in economics deems economic agents as aiming exclusively at personal material outcomes and acting as flawless maximizers. This conceptualization has been dubbed homo oeconomicus (Thaler and Mullainathan, 2001). Decades of research in psychology and economics, however, have successfully challenged this view: a large body of experimental evidence has showed that many people also respond to motivations that go beyond the maximization of personal material outcomes (Fehr and Schmidt, 1999; Bolton and Ockenfels, 2000; Andreoni and Miller, 2002; Charness and Rabin, 2002; Engelmann and Strobel, 2004). Furthermore, it has pointed out various limitations in individual rationality (e.g., Tversky and Kahneman, 1974; Nagel, 1995; Simon, 1955; Conlisk, 1996; Blume and Easley, 2008).

This study is about how pro-social motivations relate to strategic reasoning. Our hypothesis is that pro-social dispositions in the population are independent from levels of strategic reasoning. We refer to this as the orthogonality hypothesis (Bostrom, 2012), which implies that naïve and sophisticated subjects exhibit on average similar degrees of pro-sociality. If supported, it would legitimize the parallel investigation of pro-social motivations in economic choices vs. bounds to rationality in the forms of strategic reasoning. Hence, this study may carry a practical implication for academic research on pro-social dispositions and strategic reasoning. However, in the alternative hypothesis that these two traits were systematically correlated, theoretical and empirical research may benefit from understanding how they relate.

To test this hypothesis, we take individual measures of some well-defined pro-social motivations and study their link with the level of strategic reasoning in an experiment with students. To study pro-sociality we employ two dictator games (Engelmann and Strobel, 2004) and to measure strategic reasoning we use a guessing game (Nagel, 1995). Dictator games are appropriate for our purpose because they enable us to identify specific aspects of pro-social preferences while controlling for strategic reasoning. In such games, strategic considerations play no role because only one subject decides and the other persons are passive recipients. Moreover, through a guessing game one can explore strategic reasoning independently from pro-social concerns, because the total group earnings remain the same irrespective of participants choices, and because the predicted equilibrium strategy is identical for self-regarding and for pro-social participants.

In the literature, strategic reasoning has been studied in relation to cognitive skills and the findings mostly suggest that it correlates with many other measures of rationality. There is experimental evidence that subjects who choose lower numbers in guessing games exhibit higher scores in a psychometric test of cognitive ability (Burnham et al., 2009), better performance in a Raven test (Gill and Prowse, 2014), are better at learning tasks (Agranov et al., 2013), exhibit higher scores in a Cognitive Reflection Test (Brañas-Garza et al., 2012; Carpenter et al., 2013), and score higher on the SAT and ACT (Carpenter et al., 2013).

Some experiments studied other dimensions of strategic reasoning: making few reasoning errors in a beauty contest game appears positively related to the performance on the Operation Span test (Rydval et al., 2009). Instead, Georganas et al. (2014) report no significant relationship between level-k playing and scores in various cognitive tests: IQ, Eye Gaze test for adult autism, Wechsler digit span working memory test, and Cognitive Reflection Test. Coricelli and Nagel (2009) find that strategic reasoning is possibly a separate and independent ability from computational skills.

The relationship between some of these cognitive abilities and pro-social dispositions has received some attention in the literature. Choices in dictator games have generally been put into relation with performance in tests of cognitive skills of various types (post-session survey, school math scores, Raven Progressive Matrix test, test of cognitive load, SAT). While some studies report that dictators with high cognitive skills give lower amounts to recipients (Millet and Dewitte, 2007; Chen et al., 2013), others report the opposite (Brandstatter and Guth, 2002; Ben-Ner et al., 2004; Benjamin et al., 2013), or find unclear effects (Hauge et al., 2009).

To the best of our knowledge, this is among the first contributions that studies the relationship between strategic reasoning using choices in a guessing game and pro-sociality in a dictator game with three players. The two closest studies are Bayer and Renou (2011) and Dittrich and Leipold (2014). The former studies the relationship between strategic reasoning using incentivized written accounts describing the reasoning behind choices instead of actual choices in the guessing game and choices in a standard two-person dictator and a trust game. They find that subjects following higher steps of reasoning are less pro-social than subjects following zero steps. Bayer and Renou (2011) employ a Dirty Faces Game to elicit the level of strategic reasoning and a dictator game to control for pro-sociality. Their focus is on how subjects play strategic-form games and do not report the direct correlation between strategic reasoning and pro-sociality. The present study yields two original results. First, it provides a more complete mapping of pro-sociality by expanding the scope of pro-social motivations under study. The standard two-person dictator game only captures the level of generosity, the degree of aversion to advantageous inequality (Fehr and Schmidt, 1999). In our modified dictator games, we elicit individual measures about the concern for advantageous and disadvantageous inequality as well as of concerns for efficiency—the willingness to give up personal earnings in order to increase overall group earnings. Second, it focuses on strategic reasoning, which is elicited in an incentivized way. We find that there is no systematic relationship between pro-sociality and strategic reasoning: Subjects appears to be distributed independently across these two dimensions as suggested by the orthogonality hypothesis.

This study leaves out some pro-social motivations such as positive and negative reciprocity or guilt aversion (Levine, 1998; Ghidoni and Ploner, 2014). Although these are important behavioral considerations, we have not included these motivations because they contain both pro-social and strategic aspects. Hence, we report here only some measures of pro-sociality and compare them with strategic reasoning. The paper is structured as follows. Section 2 describes the experimental design, Section 3 reports results, and Section 4 discusses results and concludes.

## 2. Materials and methods

We recruited 195 students from Purdue University using in-class announcements and then asking them to sign-up online through the ORSEE platform (Greiner, 2004). A session consisted of three parts. The first part consisted of two tasks aimed at eliciting pro-social preferences along the lines of Engelmann and Strobel (2004). The second part included a guessing game (Nagel, 1995) aimed at assessing participants depth of strategic reasoning. The third part comprises a series of modified trust games. In a companion paper—Arruñada and Casari (2013)—we report on the third part and employ part one and two simply as controls. There we do not discuss the results of the first two parts, which is done here. Complete instructions including part three are available upon request to the authors.

At the outset, every participant had to submit a choice in each one of the two dictator games described in Table 1. Each dictator game proposed a choice between alternative allocations of money within a group of three: Person 1, Person 2, and Person 3. The dictator (Person 2) decided among three available options: A, B, and C in game 1 and D, E, and F in game 2. Choices were made though pen-and-paper.

**Table 1:** **Dictator and Guessing games**.

**Dictator games**						

	**(1) Equality vs. Self-regard**			**(2) Efficiency vs. Self-regard**		
	**A**	**B**	**C**	**D**	**E**	**F**
Person 1	8	11	12	20.5	12	7.5
Person 2 (dictator)	8	8.5	9	6.5	7	7.5
Person 3	8	4.5	3	5	5	5
Total payoffs	24	24	24	32	24	20
*Predictions:*						
Self-regard			x			x
Efficiency	x	x	x	x		
Inequality aversion	x					x
Maximin	x			x	x	x
*Experimental choices (N = 195)*						
N of participants	94	27	74	100	32	63
Percentage	48.2	13.8	37.9	51.3	16.4	32.3
**Guessing game**	(min 0, max 100)					
*Predictions:*	0 = Nash equilibrium					
*Experimental choices (N = 195)*						
Mean guess	42.7	39.0	39.7	39.6	44.6	41.6
Median guess	38.0	36.0	37.5	35.5	41.6	42.0

We followed the strategy method. Every participant made choices as if she was the dictator: she should imagine to be Person 2 and assign to the other two members of the group the earnings related to Person 1 and Person 3 as reported in Table 1. All participants wrote their choices on a decision card that was marked with their anonymous identification number. The experimenter collected the cards, shuffled them, randomly formed groups, and followed the procedure described below to determine the individual payments. In each group, the decision card of one member was randomly selected to be Person 2 and the other two were assigned to Person 1 and 3. The card of the participant that was randomly selected as Person 2 was used to define the earnings in the group. Participants were informed that their decision would be implemented only if their card was randomly selected to be Person 2. The choices of the participant selected as Person 1 and Person 3 had no impact on the outcome. Half of the groups were paid according to choices made for the first dictator game and the other half to choices related to the second dictator game.

Table 1 lists the predictions according to various criteria. The predicted outcomes for inequality aversion are coherent with two widely employed economic models of pro-sociality (Engelmann and Strobel, 2004).

To measure depth of strategic reasoning, we then introduced a one-shot guessing game. All participants had to write a real number between 0 and 100 on their personal decision cards. They were informed that groups of three would be randomly formed, and then a target number for each group would be computed by taking two thirds of the group average. Within each group, the participant closest to her target number received 6 points, which were evenly split in case of a tie. After every participant made a choice, the experimenter collected all decision cards and wrote the results for both tasks on the cards, and returned them to the participants who learned the results of these tasks at the end of the session.

Earnings were paid privately at the end of a session summing the points of all parts of the experiment: cash payments varied depending on the outcome according to the publicly announced rate of 0.45 USD for every point earned. Experimental instructions for the first and second parts are in the Appendix. All sessions were conducted in conformity with the relevant US regulatory standards. In particular, this study has obtained the approval of the IRB committee of Purdue University.

Each participant was present in only one session. There were a total of 16 sessions. A session included between 6 and 18 subjects. A session lasted on average less than 2 h (this also includes the third part of the experiment), including instruction reading. A participant earned on average $24.

In dictator game 1, the best choices for a self-regarding agent is option C. Choosing A or B reveals a willingness to forgo personal earnings to achieve a pro-social outcome: this would show a concern for equality. In dictator game 2, F maximizes the dictators earnings. Choosing D or E reveals a willingness to sacrifice personal earnings to increase group overall earnings, which indicates a concern for efficiency—the pro-social outcome. We have identified the best choice according to some criteria that the subjects may want to follow (Table 1). These criteria include two widely employed economic models of pro-sociality, which codify inequality averse preferences (see Engelmann and Strobel, 2004 for a discussion).

The guessing game has a unique Nash equilibrium. It can be found by the iterated deletion of dominated strategies. This solving procedure predicts that a sophisticated player never chooses values larger than 66.66 (= 100 × 2/3), no matter what others chose. If everyone does so, then strategic reasoning would suggest to eliminate values larger than 44.44 (= 100 × (2/3)^2^). Further iterations of this type of reasoning, will lead everyone to choose 0, which is the Nash equilibrium.

## 3. Results

In this Section we present the raw choices separately for each task—dictator and guessing games—and then in relation to one another. In the dictator game about 37.9% of the participants chose C and 32.3% chose F (Table 1): these choices reveal that only a minority of participants fit the predictions of a self-regarding agent. These results are in line with previous studies reporting the pro-sociality dispositions of a sizable share of the population (e.g., Güth et al., 2001; Andreoni and Miller, 2002).

**Figure 1:**
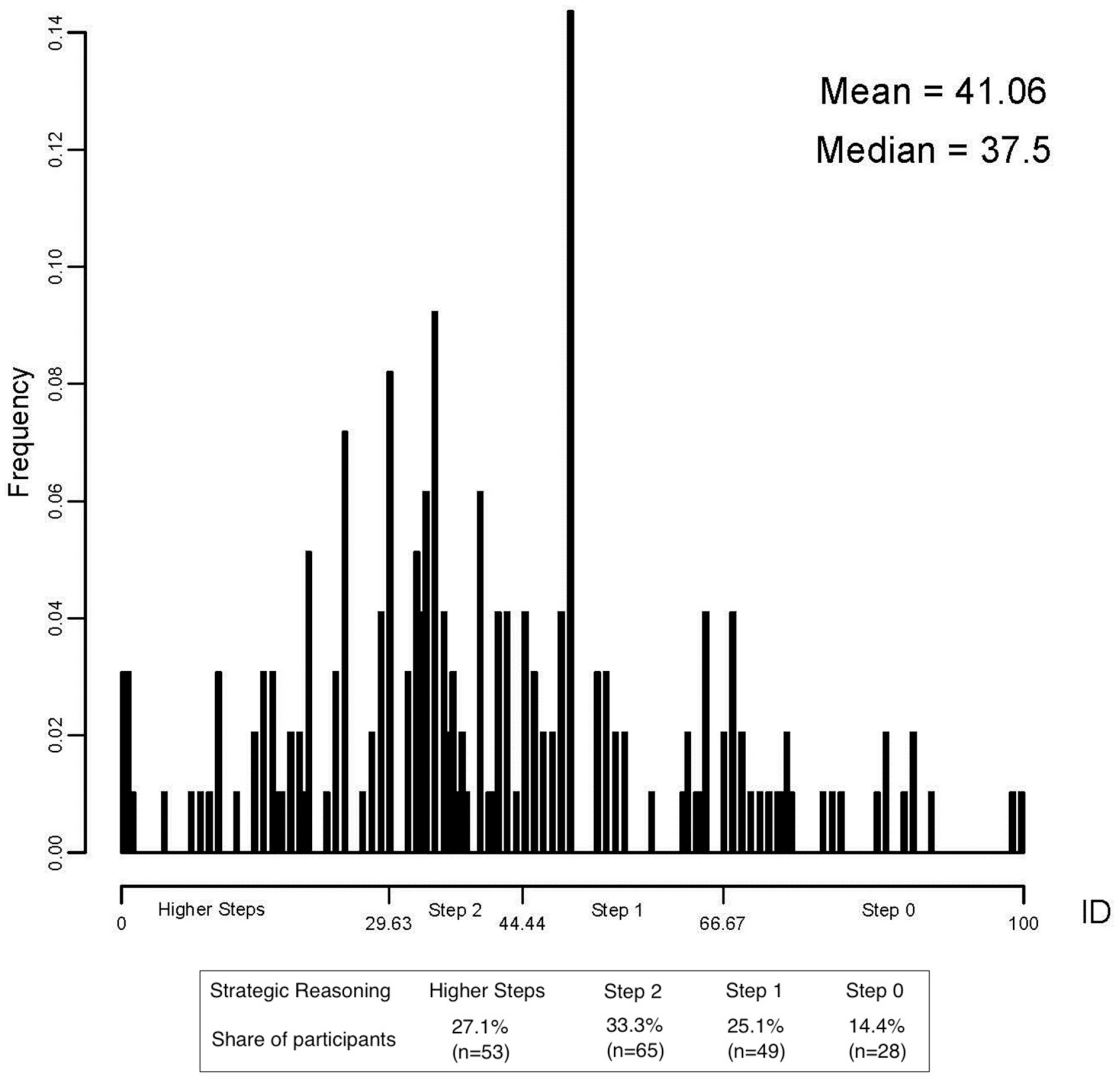
**Distribution of choices in the guessing game**.

On the guessing game, individual guesses thoroughly spread from 0 through 100 (Figure 1). Other experiments with the guessing game adopted multipliers of 1/2 or 2/3 and obtained similar results (e.g., Nagel, 1995; Coricelli and Nagel, 2009). Note that the same equilibrium of zero holds for any game with a multiplier strictly below 1. We report data about choices in the guessing game classified according to the implicit number of steps of reasoning as in an Iterated Dominance solution model (ID, see Bosch-Domenech et al., 2002).

To study the relationships between levels of strategic reasoning and pro-sociality, we compared the revealed level of pro-sociality and the patterns of guesses.

**Figure 2:**
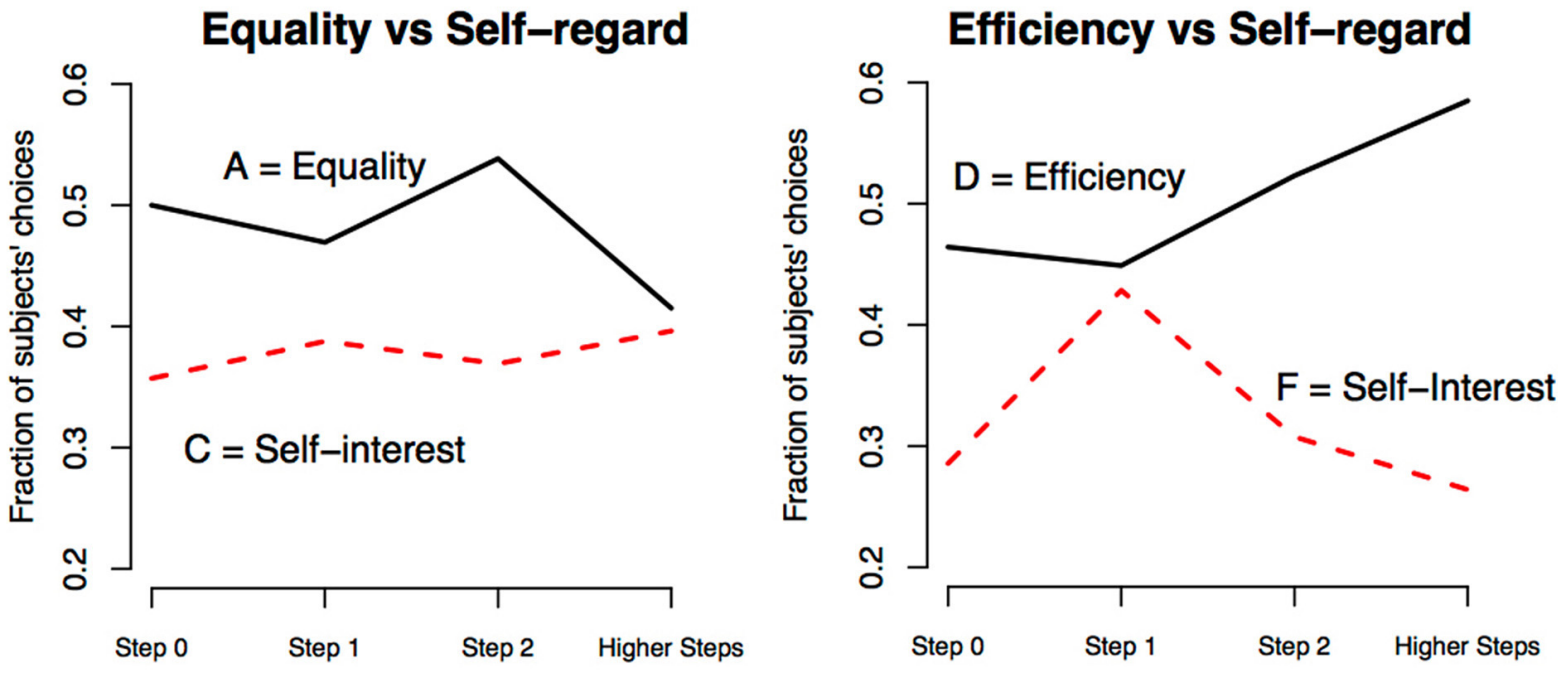
**Pro-social concerns and steps of strategic reasoning**. Fraction of participants choosing A(C) or D(F) in dictator games computed separately on clusters of participants classified in the same step of reasoning.

In Figure 2 and in Table 2 we represent the fraction of subjects choosing options A and C in the dictator game subdivided by steps of reasoning in the guessing game.

**Table 2:** **Joint choices in dictator and guessing games (number of participants)**.

	**Step 0**	**Step 1**	**Step 2**	**Higher steps**	
	**(66.66–100)**	**(44.44–66.66)**	**(29.63–44.44)**	**(0–29.63)**	**Totals**
**DICTATOR GAME 1**					
A	14	23	35	22	94
B	4	7	6	10	27
C	10	19	24	21	74
**DICTATOR GAME 2**					
D	13	22	34	31	100
E	7	6	11	8	32
F	8	21	20	14	63
Totals	28	49	65	53	195

In the left panel one can see that those who chose the pro-social option A and those who chose the self-regarding option C in dictator game 1 are a roughly constant share in each step of reasoning. In the right panel one can see that those who chose option D vs. F in game 2 do not vary systematically across step of reasoning. These findings are supported by a series of probit regressions, which show a lack of significant pro-sociality differences among decision makers characterized by distinct steps of reasoning (Table 3). The dependent variable of these regressions takes value 1 when the participant chose a specific option in a dictator game (A, C, D, or F). The regressors are a list of dummy variables that codify the number of steps of reasoning according to the ID model. As a robustness check, one specification contrasts Step 0 vs. more than 0, another separate Step 0 from Step 1 and cluster Step 2 or higher, while another considers all four step category separately. In each of these three econometric specifications—within the four options A, C, D, and F—we do not find a relationship between pro-sociality and strategic reasoning.

**Table 3:** **Pro-social preferences and strategic reasoning**.

**Dep. variable**	**Choice for self-regard(C)**			**Choice for equality (A)**			**Choice for self-regard (F)**			**Choice for efficiency (D)**		
Step 1	0.081	0.081		−0.077	−0.077		0.386	0.386		−0.039	−0.039	
	(0.303)	(0.303)		(0.297)	(0.297)		(0.309)	(0.309)		(0.298)	(0.298)	
Step 2	0.032			0.097			0.064			0.148		
	(0.290)			(0.283)			(0.299)			(0.284)		
Higher Steps	0.103			−0.214			−0.065			0.304		
	(0.299)			(0.294)			(0.312)			(0.294)		
Step 2 or Higher		0.064			−0.042			0.007			0.217	
		(0.270)			(0.263)			(0.279)			(0.264)	
Step 1 or Higher			0.069			−0.053			0.124			0.142
			(0.262)			(0.256)			(0.271)			(0.256)
Constant	−0.366	−0.366	−0.366	0.000	0.000	0.000	−0.566^**^	−0.566^**^	−0.566^**^	−0.090	−0.090	−0.090
	(0.243)	(0.243)	(0.243)	(0.237)	(0.237)	(0.237)	(0.251)	(0.251)	(0.251)	(0.237)	(0.237)	(0.237)
Log likelihood	−129.360	−129.408	−129.405	−134.111	−135.004	−135.001	−120.934	−121.069	−122.581	−133.999	−134.225	−134.954

Probit models with regressors dummy variables for the Steps of reasoning of the ID model. The default value of strategic reasoning is Step 0. Coefficient estimates with standard errors in parentheses are reported. Statistical significance: ^***^1%,

** *5%*,

^*^10%. Number of observations is 195 in all models.

One can also jointly consider the two choices in the dictator games. This allows us to identify strictly self-regarding participants as those who chose C and F (*n* = 42, 21.5%), and strongly pro-social as those who chose A and D (*n* = 63, 32.3%). These two categories of participants are less likely to be classified as self-regarding or pro-social by pure chance. A series of probit regressions on the joint choices generally reveals no correlation between being self-interest or pro-social and the number of steps of reasoning (Table 4). An exception is the higher fraction of self-regarding choices for those classified as Step 1 reasoning over Step 0, which is weakly significant (see columns (1) and (2) in Table 4). In addition, we performed a variety of robustness checks with different specifications that include as additional observations the intermediate choices (B, E), or employ another set of regressors for the level of strategic reasoning, or follow an alternative classification of individuals in terms of strategic reasoning (for instance the Iterated Best Reply model, as of Nagel, 1995, see Online Supplementary Material).

**Table 4:** **Pro-social preferences and strategic reasoning (combined)**.

**Dep. variable**	**Choice for self-regard (C and F)**			**Choice for pro-social (A and D)**		
Step 1	0.676^*^	0.676^*^		−0.061	−0.061	
	(0.369)	(0.369)		(0.316)	(0.316)	
Step 2	0.454			0.313		
	(0.362)			(0.296)		
Higher steps	0.427			0.047		
	(0.372)			(0.310)		
Step 2 or Higher		0.442			0.197	
		(0.342)			(0.278)	
Step 1, 2 or Higher			0.515			0.124
			(0.334)			(0.271)
Constant	−1.242^***^	−1.242^***^	−1.242^***^	−0.566^**^	−0.566^**^	−0.566^**^
	(0.317)	(0.317)	(0.317)	(0.251)	(0.251)	(0.251)
Log-likelihood	−99.780	−99.786	−100.300	−121.305	−121.922	−121.581

*Probit models with regressors dummy variables for the Steps of reasoning of the ID model. The default value of strategic reasoning is Step 0. Coefficient estimates with standard errors in parentheses are reported. Statistical significance*:

*** *1%*,

** *5%*,

* *10%*.

*Number of observations is 195 in all models*.

To sum up, the evidence outlined in this Section supports two main results.

Result 1. *The fraction of participants who prefer equality over self-regard is similar across steps of reasoning*.

Result 2. *The fraction of participants who prefer efficiency over self-regard is similar across steps of reasoning*.

## 4. Discussion

In this paper we classify participants according to a proxy of their depth of strategic reasoning in a guessing game, and then considered their pro-social dispositions as revealed in two dictator games. The evaluation of pro-sociality considers both concerns for equality and efficiency, which are aspects that currently play a central role in the social sciences within the debate about motivations for economic behavior (Fehr and Schmidt, 1999; Bolton and Ockenfels, 2000; Andreoni and Miller, 2002; Charness and Rabin, 2002; Engelmann and Strobel, 2004; Rand et al., 2012).

We report that the level of strategic reasoning has no significant relationship with pro-sociality. In particular, participants with a low ability to perform strategic reasoning exhibit similar degrees of pro-sociality than others, which suggests that deviations from the self-regarding behavior appears to be a purposeful action. One practical implication of this result is that scholars can proceed in parallel in the investigation of pro-social motivations in economic choices vs. bounds to rationality in the forms of strategic reasoning.

Arguably, strategic reasoning is only a specific dimension within the assumption of rationality in economic behavior. As suggested by an anonymous referee, by focusing on game-theoretical procedures to solve the game, we provide a computational account of the cognitive process underlying the actual decision-making. A more systematic test of the initial conjectures could include also other dimensions of rationality, such as numeracy ability, IQ, ability to deal with cognitive loads, consistency in choices, which are not explored in this paper.

The implications of our findings are at the moment limited to strategic reasoning and not to the more encompassing assumption of rationality. Although somewhat specific, strategic reasoning is a cognitive process with wide applications in economic settings. Whenever individuals are asked to reason iteratively and think inductively, strategic reasoning is at play (Carpenter et al., 2013). Consider for instance the choice to save for retirement or how much to invest in education. Moreover, when others' behavior is relevant for personal outcome, because interaction is at play, both personal strategic ability and the ability to assess the strategic sophistication of others is crucial.

An important qualification related to the ability to predict others' levels of reasoning is required in our design. The classification of participants by depth of reasoning presents indeed limitations: according to the Iterative Dominance model (ID), while any choice equal or above 66.7 is a clear sign of lack of strategic reasoning, any choice less than 66.7 can be rationalized, by assuming a specific belief about what others will do. As we do not observe individual beliefs, we cannot rule out those values as unreasonable. Hence, a high guess either characterizes participants with low steps of reasoning or those who believe that everybody else is unable perform strategic reasoning (Coricelli and Nagel, 2009).

We interpret the data according to the former view and hence assume that there is a positive correlation between the level of strategic reasoning of a person and her belief about the level of strategic reasoning of the others. Finally, the assessment of the individual level of strategic reasoning is tied to the use of a particular model, in this case the ID model. Alternative classifications in steps are possible and codify different reasoning protocols, for instance the Iterated Best Reply model (IBR, see Nagel, 1995). Nevertheless, our results do not substantially change if the IBR model is used (see Online Supplementary Material). Possible robustness checks for this result include using an improved classification in steps of reasoning that employs elicited self-assessment of individual reasoning process, beliefs, or other information. We leave this to future research.

In summary, our evidence suggests no direct relationship between pro-social concerns and the ability to perform strategic reasoning.

### Conflict of interest statement

The authors declare that the research was conducted in the absence of any commercial or financial relationships that could be construed as a potential conflict of interest.
