# Supplementary material for: Pro-sociality and strategic reasoning in economic decisions
Source: Front Behav Neurosci. 2015 May 28;9:140. doi: 10.3389/fnbeh.2015.00140 (PMC4446529; doi:10.3389/fnbeh.2015.00140)
Supplement: Supplementary file 1 [file Table1.PDF]

Pro-sociality and Strategic Reasoning in Economic Decisions Supplementary Online Material

Benito Arruñada, Marco Casari and Francesca Pancotto*

May 8, 2015

*Corresponding author: Pancotto, Universita' di Modena and Reggio Emilia, Department of Comunication and Economics, Reggio Emilia, email: francesca.pancotto@unimore.it.

A Additional Tables

Table A1: Pro-social preferences and strategic reasoning (Step 1 reference Step)

Dep. Variable	Choice for Self-regard (C)		Choice for Equality (A)		Choice for Self-regard (F)		Choice for Efficiency (D)	
Step 0	-0.081	-0.081	0.077	0.077	-0.386	-0.386	0.039	0.039
	(0.303)	(0.303)	(0.297)	(0.297)	(0.309)	(0.309)	(0.298)	(0.298)
Step 2	-0.049		0.173		-0.322		0.186	
	(0.241)		(0.237)		(0.243)		(0.238)	
Higher Steps	0.022		-0.138		-0.451*		0.343	
	(0.252)		(0.250)		(0.258)		(0.250)	
Step 2 or Higher		-0.017		0.034		-0.379*		0.256
		(0.216)		(0.213)		(0.218)		(0.214)
Constant	-0.285	-0.285	-0.077	-0.077	-0.180	-0.180	-0.128	-0.128
	(0.182)	(0.182)	(0.179)	(0.179)	(0.180)	(0.180)	(0.180)	(0.180)
Log-likelihood	-129.36	-129.405	-134.111	-135.004	-120.934	-121.069	-133.999	-134.225

Notes: Probit models with regressors dummy variables for the Steps of reasoning of the ID model. The default value of strategic reasoning is Step 1. Coefficient estimates with standard errors in parentheses are reported. Statistical significance: $***$ 1%, $**$ 5%, $*$ 10%. Number of observations is 195 for all models.

Table A2: Pro-social preferences and strategic reasoning (Higher Steps only)

Dep. Variable	Choice for Self-regard (C)	Choice for Equality (A)	Choice for Self-regard (F)	Choice for Efficiency (D)
Higher Steps	0.060	-0.232	-0.232	0.250
	(0.205)	(0.203)	(0.215)	(0.203)
Constant	-0.323***	0.018	-0.399***	-0.035
	(0.107)	(0.105)	(0.108)	(0.105)
Log-likelihood	-129.400	-134.381	-122.096	-134.339

Notes: Probit models with regressors dummy variables for the Steps of reasoning of the ID model. Coefficient estimates with standard errors in parentheses are reported. Statistical significance: $***$ 1%, $**$ 5%, $*$ 10%. Number of observations is 195 for all models.

Table A3: Pro-social preferences and strategic reasoning (Without Steps of reasoning - Guess as continuous variable)

Dep. Variable	Choice for Self-regard (C)	Choice for Equality (A)	Choice for Self-regard (F)	Choice for Efficiency (D)
Guess	-0.003	0.005	0.001	-0.004
	(0.004)	(0.004)	(0.005)	(0.004)
Constant	-0.177	-0.238	-0.505**	0.210
	(0.202)	(0.200)	(0.208)	(0.199)
Log-likelihood	-129.185	-134.452	-122.657	-134.594

Notes: Probit model with dependent variables the choices A and C and D and F and as regressors the value of the choice in the guessing game. Coefficient estimates with standard errors in parentheses are reported. Statistical significance: $***$ 1%, $**$ 5%, $*$ 10%. Number of observations is 195 for all models..

Table A4: Pro-social preferences and strategic reasoning (Separate Steps of reasoning)

Dep. Variable	Choice for Self-regard (C)	Choice for Pro-social (A)	Choice for Self-regard (F)	Choice for Efficiency (D)
Step 0	0.081	-0.077	0.386	-0.039
	(0.303)	(0.297)	(0.309)	(0.298)
Step 1	0.032	0.097	0.064	0.148
	(0.290)	(0.283)	(0.299)	(0.284)
Step 3	0.366	-0.396	-0.049	0.284
	(0.345)	(0.347)	(0.364)	(0.343)
Step 4	-1.017*	0.674	-0.401	0.520
	(0.574)	(0.459)	(0.498)	(0.443)
Step 5	0.282	-0.623	0.135	0.173
	(0.405)	(0.421)	(0.419)	(0.402)
Constant	-0.366	0.000	-0.566**	-0.090
	(0.243)	(0.237)	(0.251)	(0.237)
Log-likelihood	-125.601	-130.360	-120.434	-133.745

Notes: Probit models with as regressors dummy variables for the Steps of reasoning of the ID model. Coefficient estimates with standard errors in parentheses are reported. Statistical significance: $***$ 1%, $**$ 5%, $*$ 10%. Number of observations is 195 for all models..

Table A5: Pro-social preferences and strategic reasoning (combined)- Replicate Table 4 including middle choice in the dictator

Dep. Variable	Choice for Self-regard (B+C and E+F)			Choice for Pro-social (A+B and D+E)		
Step 1	0.070	0.070		-0.676*	-0.676*	
	(0.308)	(0.308)		(0.369)	(0.369)	
Step 2	0.047			-0.454		
	(0.294)			(0.362)		
Higher Steps	-0.055			-0.427		
	(0.306)			(0.372)		
Step 2 or Higher		0.002			-0.442	
		(0.274)			(0.342)	
Step 1, 2 or Higher			0.022			-0.515
			(0.266)			(0.334)
Constant	-0.464*	-0.464*	-0.464*	1.242***	1.242***	1.242***
	(0.246)	(0.246)	(0.246)	(0.317)	(0.317)	(0.317)
Log-likelihood	-123.274	-123.363	-123.412	-99.780	-99-786	-100.300

Notes: Probit model with dependent variables the choices A and C and D and F and as regressors dummy variables for the Steps of reasoning of the ID model. The default value of strategic reasoning is Step 0. Coefficient estimates with standard errors in parentheses are reported. Statistical significance: $***$ 1%, $**$ 5%, $*$ 10%. Number of observations is 195 for all models.

Table A6: Pro-social preferences and strategic reasoning - replicate Table 3 with IBR model

Dep. Variable	Choice for Self-regard (C)			Choice for Equality (A)			Choice for Self-regard (F)			Choice for Efficiency (D)		
Step 1	0.238	0.238		-0.348	-0.348		0.242	0.242		-0.060	-0.060	
	(0.230)	(0.230)		(0.227)	(0.227)		(0.231)	(0.231)		(0.226)	(0.226)	
Step 2	0.161			-0.178			-0.250			0.301		
	(0.266)			(0.262)			(0.280)			(0.263)		
Higher Steps	-0.083			-0.252			-0.298			0.315		
	(0.276)			(0.266)			(0.288)			(0.268)		
Step 2 or Higher		0.045			-0.214			-0.273			0.308	
		(0.224)			(0.219)			(0.233)			(0.219)	
Step 1, 2 or Higher			0.136			-0.276			-0.019			0.137
			(0.198)			(0.194)			(0.201)			(0.194)
Constant	-0.401**	-0.401**	-0.401**	0.144	0.144	0.144	-0.446***	-0.446***	-0.446***	-0.062	-0.062	-0.062
	(0.165)	(0.165)	(0.165)	(0.161)	(0.161)	(0.161)	(0.166)	(0.166)	(0.166)	(0.161)	(0.161)	(0.161)
Log-likelihood	-128.498	-128.822	-129.207	-133-808	-133.840	-134.028	-120.098	-120.109	-122.682	-133.428	-133.429	-134.850

Notes: Probit models with regressors dummy variables for the Steps of reasoning of the IBR model. The default value of strategic reasoning is Step 0. Coefficient estimates with standard errors in parentheses are reported. Statistical significance: $***$ 1%, $**$ 5%, $*$ 10%. Number of observations is 195 in all models.

Table A7: Pro-social preferences and strategic reasoning (combined). Replicate Table 4 with IBR model

Dep. Variable	Choice for Self-regard (C and F)			Choice for Pro-social (A and D)		
Step 1	0.253	0.253		-0.248	-0.248	
	(0.250)	(0.250)		(0.239)	(0.239)	
Step 2	-0.027			0.231		
	(0.301)			(0.265)		
Higher Steps	-0.095			-0.165		
	(0.311)			(0.279)		
Step 2 or Higher		-0.060			0.045	
		(0.252)			(0.224)	
Step 1, 2 or Higher			0.094			-0.085
			(0.220)			(0.200)
Constant	-0.853***	-0.853***	-0.853***	-0.401**	-0.401**	-0.401**
	(0.184)	(0.184)	(0.184)	(0.165)	(0.165)	(0.165)
Log-likelihood	-100.644	-100.664	-101.504	-120.923	-121.768	-122.597

Notes: Probit models with regressors dummy variables for the Steps of reasoning of the IBR model. The default value of strategic reasoning is Step 0. Coefficient estimates with standard errors in parentheses are reported. Statistical significance: $***$ 1%, $**$ 5%, $*$ 10%. Number of observations is 195 in all models.

Table A8: Pro-social preferences and strategic reasoning (Step 0 reference Step). Replicate Table A1 with IBR model

Dep. Variable	Choice for Self-regard (C)		Choice for Equality (A)		Choice for Self-regard (F)		Choice for Efficiency (D)	
Step 0	-0.238	-0.238	0.348	0.348	-0.242	-0.242	0.060	0.060
	(0.230)	(0.230)	(0.227)	(0.227)	(0.231)	(0.231)	(0.226)	(0.226)
Step 2	-0.077		0.170		-0.492*		0.361	
	(0.263)		(0.261)		(0.277)		(0.262)	
Higher Steps	-0.321		0.096		-0.540*		0.375	
	(0.273)		(0.266)		(0.284)		(0.267)	
Step 2 or Higher		-0.193		0.134		-0.515**		0.368*
		(0.220)		(0.218)		(0.228)		(0.219)
Constant	-0.162	-0.162	-0.204	-0.204	-0.204	-0.204	-0.122	-0.122
	(0.160)	(0.160)	(0.160)	(0.160)	(0.160)	(0.160)	(0.160)	(0.160)
Log-likelihood	-128.498	-128.822	-133.808	-133.840	-120.098	-120.109	-133.428	-133.429

Notes: Probit models with regressors dummy variables for the Steps of reasoning of the IBR model. The default value of strategic reasoning is Step 0. Coefficient estimates with standard errors in parentheses are reported. Statistical significance: $***$ 1%, $**$ 5%, $*$ 10%. Number of observations is 195 in all models.

Table A9: Pro-social preferences and strategic reasoning (Higher Steps only). Replicate Table A2 with IBR model

Dep. Variable	Choice for Self-regard (C)	Choice for Equality (A)	Choice for Self-regard (F)	Choice for Efficiency (D)
Higher Steps	-0.214	-0.076	-0.341	0.269
	(0.243)	(0.234)	(0.256)	(0.236)
Constant	-0.270***	-0.031	-0.402***	-0.016
	(0.100)	(0.099)	(0.102)	(0.099)
Log-likelihood	-129.050	-134.984	-121.772	-134.446

Notes: Probit model with regressors dummy variables for the Steps of reasoning of the IBR model. Coefficient estimates with standard errors in parentheses are reported. Statistical significance: $***$ 1%, $**$ 5%, $*$ 10%. Number of observations is 195 in all models.

Table A10: Pro-social preferences and strategic reasoning (Separate Steps of reasoning. Replicate Table A3 with IBR model)

Dep. Variable	Choice for Self-regard (C)	Choice for Pro-social (A)	Choice for Self-regard (F)	Choice for Efficiency(D)
Step 1	0.238	-0.348	0.242	-0.060
	(0.230)	(0.227)	(0.231)	(0.226)
Step 2	0.161	-0.178	-0.250	0.301
	(0.266)	(0.262)	(0.280)	(0.263)
Step 3	-0.441	0.109	-0.591	0.447
	(0.360)	(0.326)	(0.381)	(0.330)
Step 4	-0.441	-0.986		0.903
	(0.660)	(0.659)	-	(0.659)
Step 5	0.654	-0.669	0.446	-0.192
	(0.434)	(0.447)	(0.430)	(0.432)
Log-likelihood	-125.951	-131.748	-116.670	-132.053

Notes: Probit models regressors dummy variables for the Steps of reasoning of the IBR model. Coefficient estimates with standard errors in parentheses are reported. Statistical significance: $***$ 1%, $**$ 5%, $*$ 10%. Number of observations is 195 in all models.

Table A11: Pro-social preferences and strategic reasoning including middle choice and IBR model

Dep. Variable	Choice for Self-regard (B+C)			Choice for Equality (A+B)			Choice for Self-regard (E+F)			Choice for Efficiency (D+E)		
Step 1	0.348	0.348		-0.238	-0.238		0.060	0.060		-0.242	-0.242	
	(0.227)	(0.227)		(0.230)	(0.230)		(0.226)	(0.226)		(0.231)	(0.231)	
Step 2	0.178			-0.161			-0.301			0.250		
	(0.262)			(0.266)			(0.263)			(0.280)		
Higher Steps	0.252			0.083			-0.315			0.298		
	(0.266)			(0.276)			(0.268)			(0.288)		
Steps 2 or Higher		0.214			-0.045			-0.308			0.273	
		(0.219)			(0.224)			(0.219)			(0.233)	
Step 1 or Higher			0.276			-0.136			-0.137			0.019
			(0.194)			(0.198)			(0.194)			(0.201)
Constant	-0.144	-0.144	-0.144	0.401**	0.401**	0.401**	0.062	0.062	0.062	0.446***	0.446***	0.446***
	(0.161)	(0.161)	(0.161)	(0.165)	(0.165)	(0.165)	(0.161)	(0.161)	(0.161)	(0.166)	(0.166)	(0.166)
Log-likelihood	-133.808	-133.840	-134.028	-128.498	-128.822	-129.207	-133.428	-133.429	-134.850	-120.098	-120.109	-122.682

Notes: Probit models regressors dummy variables for the Steps of reasoning of the IBR model. Coefficient estimates with standard errors in parentheses are reported. Statistical significance: $***$ 1%, $**$ 5%, $*$ 10%. Number of observations is 195 in all models.

Table A12: Pro-social preferences and strategic reasoning (combined) including middle choice with IBR model

Dep. Variable	Choice for Self-regard (B+C and E+F)			Choice for Pro-social (A+B and D+E)		
Step 1	0.205	0.205		-0.253	-0.253	
	(0.233)	(0.233)		(0.250)	(0.250)	
Step 2	0.110			0.027		
	(0.270)			(0.301)		
Higher Steps	-0.252			0.095		
	(0.288)			(0.311)		
Steps 2 or Higher		-0.057			0.060	
		(0.229)			(0.252)	
Step 1, 2 or Higher			0.068			-0.094
			(0.202)			(0.220)
Constant	-0.492***	-0.492***	-0.492***	0.853***	0.853***	0.853***
	(0.168)	(0.168)	(0.168)	(0.184)	(0.184)	(0.184)
Log-likelihood	-122.017	-122.679	-123.358	-100.644	-100.664	-101.503

Notes: Probit model with regressors dummy variables for the Steps of reasoning of the IBR model. The default value of strategic reasoning is Step 0. Coefficient estimates with standard errors in parentheses are reported. Statistical significance: $***$ 1%, $**$ 5%, $*$ 10%. Number of observations is 195 in all models.

B Experimental Instructions

You are participating in a decision-making experiment, where you have the opportunity to earn cash. Purdue University and other institutions have provided funds for this research. The instructions are simple. If you follow them carefully you may earn an appreciable amount of money. The actual amount of cash you will earn depends on your choices and the choices of other persons. These earnings will be paid to you in cash at the end of the experiment. Earnings are measured in points. You will be paid 45 cents ($0.45) for every experimental point you have earned in the selected round.

We ask that you do not talk with one another for the duration of the experiment. The experiment is composed of three parts. The tasks in each part are completely independent. We are now reading the instructions for part 1.

Instructions Part 1

Each participant will be assigned to one of three possible roles: ‘Person 1’, ‘Person 2’, or ‘Person 3’. The assignment is random and will be revealed at the end of the experiment.

Each Person 1 will be randomly matched with a Person 2 and a Person 3 and will form a set. You will not know the identity of the others in your set.

Your tasks consist in two simple decisions, illustrated by Table 1 and Table 2 below.

In Table 1 you have to choose the earnings of the persons in your set among options A, B, and C as if you knew you are Person 2. You actually ignore whether you are Person 1, Person 2, or Person 3 but the only case in which your decision counts is when you are selected as Person 2. There is one chance out of three that you will be selected as Person 2, and in that case your decision will be implemented. If you are assigned the role of Person 1 or Person 3, instead, your decision will not be used.

Please write your choice A, B, or C on the white card. Your decision will remain anonymous. The results will be announced at the end of the session.

In Table 2 you have to choose the earnings of the persons in your set among options D, E, and F as if you knew you are Person 2. As before you actually ignore whether you are Person 1, Person 2, or Person 3 but the only case in which your decision counts is when you are Person 2. There is one chance out of three that you will be selected as Person 2, and in that case your decision will be implemented. If you are assigned the role of Person 1 or Person 3, instead, your decision will not be used. Please write your choice D, E, or F on the white card. Your decision will remain anonymous.

Later on, we will collect all the cards with your choices, mix them, and divide them

Figure 1: Choice Tables

Table 1

	Earnings options		
	A	B	C
Person 1	8.0	11.0	12.0
**Person 2**	8.0	8.5	9.0
Person 3	8.0	4.5	3.0
Total points	24	24	24

Table 2

	Earnings options		
	D	E	F
Person 1	20.5	12.0	7.5
**Person 2**	6.5	7.0	7.5
Person 3	5.0	5.0	5.0
Total points	32	24	19

into sets of three. One random card in each set will be marked as person 2. Then for half of the sets we will pay decisions in Table 1 and for the other half decisions in Table 2. The results will be announced at the end of the session. If there is anything that you do not understand, please raise your hand. An experimenter will then come to you and clarify the problem.

Instructions Part 2

Each participant has to choose a number between zero (0) and one hundred (100). Zero and 100 are also possible. It is also possible to choose a non-integer.

You can write the number on the white card.

Later on, we will collect all the cards, mix them, and divide them into sets of three. You could be matched with any two other participants and will not know their identities.

For each set of three cards, we compute the average of the three numbers and then multiply it by 2/3. That defines the target number in the set. The person in the set whose number is closest to the target number earns 6 points. The other two earn zero. If two or three persons are equally close, the 6 points are split evenly.
